# FIGS Study Outcomes: Disentangling Relationships Between Vision Loss, the Environment, Physical Activity, and Falls

**DOI:** 10.1093/geroni/igaa057.2779

**Published:** 2020-12-16

**Authors:** Pradeep Ramulu, Laura Gitlin, Jennifer Schrack

## Abstract

The longitudinal relationships between various aspects of mobility (with each other and with vision loss) are important to understand for healthy aging. The Falls in Glaucoma Study (FIGS) was a three-year longitudinal study conducted in persons with a range of visual field damage from glaucoma (from normal visual fields to severe visual field damage) and evaluated several aspects of mobility: physical function (gait and balance), physical activity (annual accelerometer trials), fall rates (prospectively-collected falls calendars), environmental features (an in-home assessment), and fear of falling. In this symposium, we present data demonstrating that: (1) physical activity is altered by visual field damage - lowering the overall amount of physical activity, and also resulting in more fragmented activity (i.e. shorter activity bouts); (2) specific home environmental features, such as better lighting, are associated with lower rates of falls within the home; (3) specific gait and balance features increase the risk of falling, but do not explain the association between visual field damage and a higher rate of falls; (4) injurious falls, but not non-injurious falls, lead to future reductions in physical activity; and (5) worsening of fear of falling (FoF) leads to either a higher rate of falls (at low FoF levels) or decreases in physical activity (at higher FoF levels). Study findings will educate the audience about the types of mobility problems found in persons with visual field damage, potential methods to prevent falls in older adults, and factors likely to predict future mobility deficits in older adults.

